# Silver Decorated and Graphene Wrapped Polypyrrole@Ni(OH)_2_ Quaternary Nanocomposite for High Performance Energy Storage Devices

**DOI:** 10.3390/polym15051267

**Published:** 2023-03-02

**Authors:** Rashida Jafer, Sarah A. Alsufyani, Javed Iqbal, Mohammad Omaish Ansari, Arshid Numan, Shahid Bashir, P. M. Z. Hasan, S. Wageh

**Affiliations:** 1 Department of Physics, Faculty of Science, King Abdulaziz University, Jeddah 21589, Saudi Arabia; 2 Center of Nanotechnology, King Abdulaziz University, Jeddah 21589, Saudi Arabia; 3 Graphene and Advanced 2D Materials Research Group, School of Engineering and Technology, Sunway University, No. 5, Jalan Universiti, Bandar Sunway, Petaling Jaya 47500, Malaysia; 4 Sunway Materials Smart Science & Engineering (SMS2E) Research Cluster, Sunway University, No. 5, Jalan Universiti, Bandar Sunway, Petaling Jaya 47500, Malaysia; 5 Higher Institution Centre of Excellence (HICoE), UM Power Energy Dedicated Advanced Centre (UMPEDAC), Level 4, Wisma R&D, Universiti Malaya, Jalan Pantai Baharu, Kuala Lumpur 59990, Malaysia

**Keywords:** energy storage, supercapattery, quaternary nanocomposite, graphene, polypyrrole, nickel hydroxide

## Abstract

In this work, silver (Ag) anchored over graphene (GN) wrapped polypyrrole (PPy)@ nickel hydroxide (Ni(OH)_2_) nanocomposites were synthesized through a combination of oxidative polymerization and hydrothermal processes. The synthesized Ag/GN@PPy–Ni(OH)_2_ nanocomposites were characterized for their morphological characteristics by field emission scanning electron microscopy (FESEM), while the structural investigations were done by X-ray diffraction and X-ray photoelectron spectroscopy (XPS). The FESEM studies showed Ni(OH)_2_ flakes and silver particles attached over the surface of PPy globules, along with the presence of GN sheets and spherical silver particles. The structural analysis also showed the presence of constituents, i.e., Ag, Ni(OH)_2_, PPy, GN, and their interaction, therefore vouching that the synthesis protocol is efficacious. The electrochemical (EC) investigations were done in potassium hydroxide (1 M KOH) using a three electrode setup. The quaternary Ag/GN@PPy–Ni(OH)_2_ nanocomposite electrode showed the highest specific capacity of 237.25 C g^−1^. The highest electrochemical performance of the quaternary nanocomposite is associated with the synergistic/additional effect of PPy, Ni(OH)_2_, GN, and Ag. The assembled supercapattery with Ag/GN@PPy-Ni(OH)_2_ as a positive and activated carbon (AC) as a negative electrode displayed eminent energy density of 43.26 Wh kg^−1^ with the associated power density of 750.00 W kg^−1^ at a current density of 1.0 A g^−1^. The cyclic stability of the supercapattery (Ag/GN@PPy–Ni(OH)_2_//AC), comprising a battery-type electrode, displayed a high cyclic stability of 108.37% after 5500 cycles.

## 1. Introduction

Due to the rapid industrialization and depletion of non-renewable fuel resources, there is a huge demand to explore newer alternatives and greener energy sources [1,2,3]. Solar, wind, tidal energy, energy from recycled waste, etc., are promising eco-friendly, low-cost alternative energy sources that have led researchers to look for ways to utilize these [4,5]. One of the major problems with these sources is their weather and day/night dependency. Thus, in order to get an uninterrupted energy supply, backup machinery is integrated with renewable energy sources.

Fuel cells and batteries are commonly used storage devices, but their major limitations are their low power supply, where a large supply is needed [6]. Supercapattery, in contrast, can solve this problem, as it can give a huge burst of power supply in combination with long-term cyclic stability. Supercapattery combines battery and capacitive-type electrode materials, and the charge is stored differently in each electrode material. The battery involves redox reactions for charge storage, while the capacitive electrode stores charges by forming an electrical double layer. If the capacitive electrodes are of two different electrode materials, the supercapacitor here is termed asymmetric. The supercapattery assemblies are continuously exploited by scientists by introducing or combing newer materials, which can enhance their energy density [7].

The electrochemical evaluation of supercapattery depends on the electrode material. Carbon-based materials and conducting polymers have been widely employed in combination with metal oxides, sulfides, hydroxides, etc., as electrodes have shown excellent electrochemical phenomena [8,9,10]. Palsaniya et al. [11] showed that their polyaniline/GN/MoS_2_ electrode displayed a specific capacitance of 142.30 F g^−1^ while retaining the cyclic stability of 98.11% at a scan rate of 100 mV s^−1^. The developed hydrogel composite of polyaniline/GN/Fe_2_O_3_ by Gupta et al. [12] demonstrated a remarkable specific capacitance of 1124 F g^−1^ at 0.25 A g^−1^. Similarly, composite of GN, polypyrrole (PPy) and Cu_2_O–Cu(OH)_2_ by Asen et al. [13] showed a high specific capacitance of 997.00 F g^−1^ at a current density of 10 A g^−1^. Among conducting polymers, PPy has many advantages owing to its reversible electrochemical doping/dedoping, low cost, and long shelf life [14,15]. Among metal oxides, sulfides, and hydroxides, nickel hydroxide Ni(OH)_2_ is exciting due to its well-defined redox behavior, layered structure, and cost-effectiveness. Liu et al. [16] showed that the ultra-small Ni(OH)_2_ nanoparticles and GN sheets delivered a specific capacitance of 1717 F g^−1^ at 0.5 A g^−1^. Similarly, a polyaniline composite with Ni(OH)_2_ has been reported to possess a specific capacitance of 113.8 F g^−1^ [17]. Thus, the ternary nanocomposite of GN@PPy–Ni(OH)_2_ is expected to exhibit eminent electrochemical characteristics owing to the synergism between the constituents. In our previous reports, it was observed that the addition of Ag nanoparticles to conducting polymer composites enhances electrochemical conductivity [14]. Thus, adding Ag nanoparticles to GN@PPy–Ni(OH)_2_ can give a new class of quaternary nanocomposite with much-enhanced electrochemical features.

A simplistic synthesis of novel quaternary nanocomposites of silver anchored on GN-wrapped PPy–Ni(OH)_2_ was synthesized. The characterization of the prepared quaternary nanocomposites was done by diverse analytical techniques such as XPS, EDAX, XRD, and FESEM. The standard three-electrode cell was used for electrochemical findings, such as galvanostatic charge–discharge and cyclic voltammetry. Finally, the evaluation of the final fabricated device comprising a battery-type quaternary nanocomposite, Ag/GN@PPy-Ni(OH)_2_ as a negative electrode and AC as a positive electrode was tested in a two electrodes system by means of CV and GCD.

## 2. Experimental

### 2.1. Materials

Pyrrole monomer (M.W. 67.09; purity 98%), Polyvinylidene fluoride (M_w_ ~534,000; purity 98%), FeCl_3_ (M.W. 162.20; purity 99.99%), Ni(NO_3_)_2_·6H_2_O (M.W. 290.79; purity 98.5%), AC (M.W. 12.01), graphite flakes (M.W. 12.01), silver nitrate (M.W. 169.87; purity 99%), ascorbic acid nitrate (M.W. 1769.12; purity 99%), H_2_SO_4_ (M.W. 98.08; purity 98%), H_3_PO_4_ (85%) sodium dodecylbenzenesulfonate (SDBS) (M.W. 348.48), and acetylene black were bought from Sigma-Aldrich. Potassium permanganate (M.W. 158.03; purity 99%), N-methyl-2-pyrrolidone (NMP) (M.W. 99.13), HCl (M.W. 36.5; purity 35%), and ethanol (M.W. 46.07; purity 99.8%) were purchased from Otto chemicals (Mumbai, India). De-ionized water was used throughout the experiments.

### 2.2. Synthesis of Ag/GN@PPy–Ni(OH)_2_

Ni(OH)_2_ was prepared by a hydrothermal method, as reported elsewhere [18]. The synthesis of graphene oxide (GO) was done by following the procedure, mentioned in our previous reports [19]. Pure PPy was prepared by in situ oxidative polymerization of pyrrole monomers using an oxidizing agent such as FeCl_3_. In a distinctive process, 1 mL of pyrrole monomer was dispersed in 100 mL of water, while the solution of FeCl_3_ was prepared by dissolving 2.35 g in 100 mL of water in another beaker. In order to initiate polymerization, the oxidant solution (aq FeCl_3_) was added to the pyrrole dispersion under vigorous stirring. The solution soon turned dark black, thereby confirming the polymerization. Thus, the formed PPy settled down after centrifuging the mixture solution. Afterwards, repeated washing was done with a mixture of water and ethanol and finally dried in an air oven at 80 °C. In order to prepare Ag/GN@PPy-Ni(OH)_2_, 0.20 g of PPy and 0.20 g of Ni(OH)_2_ were mixed with 10 mL of GO solution (4 mg mL^−1^) and to AgNO_3_ (20 mg), followed by the addition of a 15 mL NH_3_ (6%) solution under stirring conditions. The whole reaction mixture was transferred into a Teflon-lined hydrothermal rector and subjected to a temperature of 150 °C for 5 h. Thus, the formed nanocomposite, Ag/GN@PPy–Ni(OH)_2_, was centrifuged in order to separate it from other impurities. After separating the synthesized material, washing was done with water and ethanol to ensure the complete removal of any impurities and finally dried in an air oven at 80 °C. The binary PPy–Ni(OH)_2_ was synthesized by mixing 0.20 g of each PPy and Ni(OH)_2_ in ethanol under stirring, followed by the evaporation of ethanol. While the ternary GN@PPy–Ni(OH)_2_ was produced by setting the hydrothermal reaction following the same conditions and procedure as mentioned above for the quaternary nanocomposite but without AgNO_3_.

### 2.3. Material Characterization

The FESEM and EDAX techniques were conducted for the topography, elemental analysis, and mapping. The XRD machine, along with Cu-*Kα* source, was used to explore the structure of the formed crystals and the identification of the phases of Ag/GN@PPy–Ni(OH)_2_ nanocomposites at 2*θ*° through a scan range of 5° to 70°. A highly evacuated (∼10^−9^ m bar) chamber, comprised of monochromatic *Al-Kα* radiation, was used to perform the XPS study.

### 2.4. Development of the Electrodes and EC studies

The positive and negative electrodes of the supercapattery were fabricated from quaternary nanocomposite of Ag/GN@PPy–Ni(OH)_2_ and AC. Chemically cleaned nickel foam of area 1 × 1 cm^2^ was used to coat active material to get the electrodes for the electrochemical measurements. The nanocomposite of Ag/GN@PPy–Ni(OH)_2_ at 75 wt. %, AC at 15 wt. %, and PVdF at 10 wt. % were mixed in NMP to prepare the slurry for the active material. The homogeneity of the solution was ensured after 12 h of magnetic stirring at ambient temperature. After a uniform drop coating of the slurry on the 1 × 1 cm^2^ nickel foam, it was left for drying in an oven at 80 °C for a period of a minimum 12 h. A similar method was implemented on GN@PPy–Ni(OH)_2_, PPy–Ni(OH)_2_ and PPy to prepare their working electrodes. The prepared electrodes were then brought to mass loading of ~5.00 ± 0.05 mg cm^−2^ of the active material. The electrode fabrication procedure was done with great accuracy to obtain consistent mass loading.

A working potential between 0–0.5 V versus reference (Ag/AgCl) was implemented to perform the cyclic voltammetry. To study the galvanostatic-charge discharge, a potential of 0.5 V versus Ag/AgCl was used through the electrochemical workstation (Gamry Interface 1010E) at a current density range of 4.0–10.0 A g^−1^.

## 3. Results and Discussion

### 3.1. Morphological Studies

**Figure 1:**
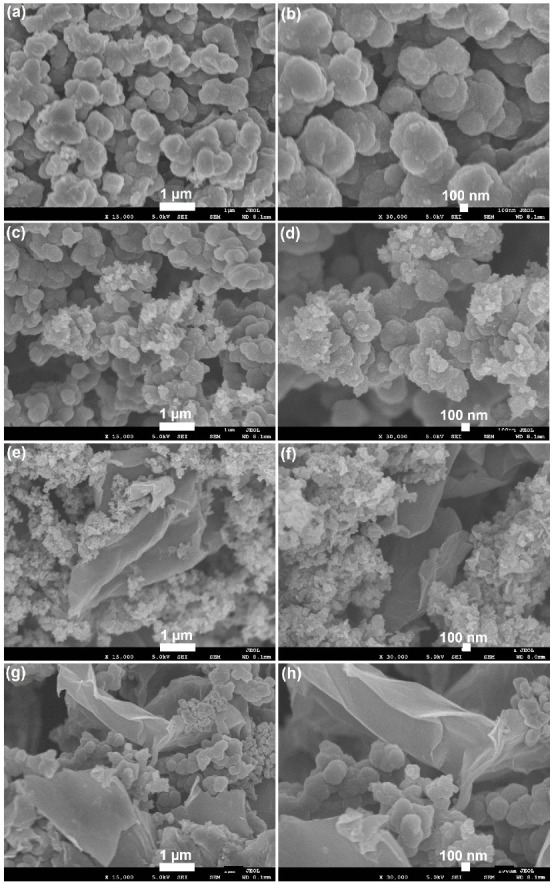
Low and high magnification micrographs of field emission scanning electron microscopy of PPy (**a**,**b**), PPy–Ni(OH)_2_ (**c**,**d**), GN@PPy–Ni(OH)_2_ (**e**,**f**), and Ag/GN@PPy–Ni(OH)_2_ (**g**,**h**).

Figure 1 shows the FESEM images of PPy, PPy–Ni(OH)_2_, GN@PPy–Ni(OH)_2_ and Ag/GN@PPy–Ni(OH)_2_ nanocomposite. Pure PPy showed loosely interconnected globular morphology with globules interconnected to form large lumps and long chains of micrometer length. The Ni(OH)_2_ flakes and particles were attached over the surface of PPy globules. It can be seen that Ni(OH)_2_ is attached as agglomerates, thereby covering PPy globules or a few clusters of Ni(OH)_2_ flaked/particles that can also be seen protruding out of PPy globules in other areas. The GN@PPy–Ni(OH)_2_ and Ag/GN@PPy–Ni(OH)_2_, in addition to the above features, show the presence of GN sheets and GN along with Ag spherical particles, respectively. The GN sheets can be seen distributed throughout the composite and the other constituents attached onto the sheets. The TEM images of Ag/GN@PPy–Ni(OH)_2_ nanocomposite at different magnifications can be seen in Figure S1.

**Figure 2:**
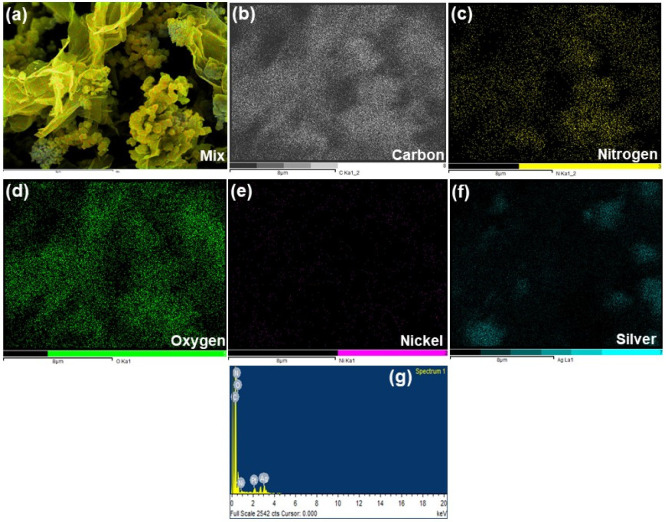
(**a**) Showing the compounded image of EDAX mapping of the quaternary nanocomposite, Ag/GN@PPy–Ni(OH)_2_, (**b**) elemental mapping of carbon contents, (**c**) nitrogen, (**d**) oxygen, (**e**) nickel, (**f**) silver, and (**g**) represents the EDAX graph.

The compounded image of EDAX mapping analysis of quaternary nanocomposite, Ag/GN@PPy–Ni(OH)_2_, is shown in Figure 2a. The uniform distribution of C, N, O, Ni, and Ag in Ag/GN@PPy–Ni(OH)_2_ could be observed from the individual elemental mapping analysis, as well as in the compounded image, which further vouched for the efficiency of the synthesis procedure (Figure 2). The image of EDAX spectra demonstrates the existence of C, N, O, Ni, and Ag. The observance of a small percentage of Pt is due to the coating of a sample used to obtain better quality results. The absence of any other peak shows the high purity of the synthesized quaternary nanocomposite.

### 3.2. Structural Studies

**Figure 3:**
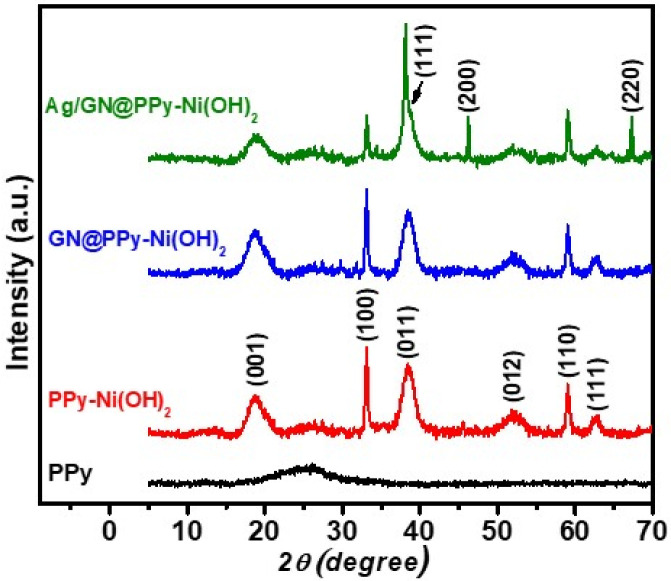
X-ray diffraction spectra of PPy, PPy−(Ni(OH)_2_, GN@PPy–Ni(OH)_2_, and Ag/GN@PPy–Ni(OH)_2_.

X−ray powder diffraction analysis was done to determine the structural and crystallinity characteristics of PPy, Ppy–Ni(OH)_2_, GN@PPy–Ni(OH)_2_, and Ag/GN@PPy–Ni(OH)_2_ nanocomposites, as shown in Figure 3. The broad, amorphous peak at ~25.00° (2*θ*°) is associated to the scattering in PPy chains at interplanar spacings [20]. PPy–Ni(OH)_2_ shows the hump of PPy, along with the peaks corresponding to Ni(OH)_2_ at 2*θ*° of 18,80° 33,10° 38.40°, 52.17°, 59.26°, and 62.87 (JCPDS 14-0117), which is in good agreement with the reports of Li et al. [21]. In the case of GN/@PPy–Ni(OH)_2_, a similar diffractogram was observed, as in the case of PPy–Ni(OH)_2_. The non-observance of graphitic 002 planes in the binary and quaternary nanocomposites’ XRD spectra suggests a few layered GN present without any graphitic impurities [22]. The Ag/GN@PPy–Ni(OH)_2_, in addition to the peaks of PPy and Ni(OH)_2_, show peaks at 2*θ* degrees of 38.71°, 48.18°, 67.44° [23], and a much-broadened peak at 2*θ*° angle of 38.40°, which might be due to the similar peak positioning of Ni(OH)_2_ and Ag.

### 3.3. X-rays Photon Electrons Spectroscopic Studies

**Figure 4:**
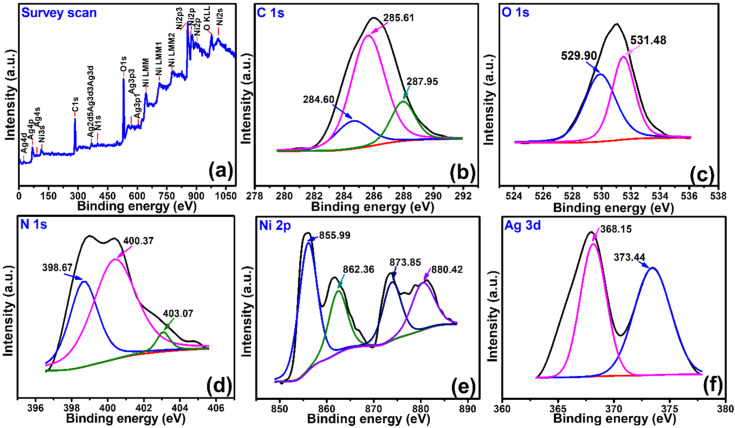
(**a**) XPS survey scan of the Ag/GN@PPy–Ni(OH)_2_ nanocomposite. The deconvoluted spectra of C1s (**b**), O1s (**c**), N1s (**d**), Ni2p (**e**), and Ag3d (**f**).

XPS studies were performed to determine the constituent elements and the functional groups in Ag/GN@PPy–Ni(OH)_2_ nanocomposites. Figure 4 depicts the survey scan, which revealed the presence of C1s, O1s, N1s, Ni2p, and Ag3d corresponding to the presence of C, O, N, Ni, and Ag, as well as the absence of any other impurities. The deconvolution of C*1s* demonstrates the presence of peaks at 284.60, 285.61, and 287.95 eV. The dominance of the 284.60 eV peak suggests the significant contributions of aliphatic C-C/C-H, while the peaks at 285.61 and 287.95 eV peaks suggest the contributions of C-O-C and O-C=O, respectively [14]. The O1s show peaks at 529.90 and 531.48 eV, corresponding to the O=N/metal bonded with oxides and C-O as well as hydroxides, respectively [24,25]. The oxygen functionalities such as carbonyl or O=N probably originated during the polymerization of pyrrole, where the chain ends react with water molecules to form oxygen functionalities [26]. Similarly, the oxidation of graphite and the ambient reduction of GO might also incorporate such oxygen functionality in GN. The N1s reveal the presence of three peaks at 398.67, 400.37, and 403.07 eV, corresponding to the amines group (-NH-) of polypyrrole ring, while the other two peaks are of oxidized/protonated nitrogen, i.e., -NH.^+^- in the polaron charge carrier species and =NH^+^-, a bipolaron charge carrier species [14]. The Ni 2p reveals two spin-orbit doublets at 855.99 and 873.85 eV, corresponding to the Ni 2p_3/2_ and Ni 2p_1/2_. The other peaks at 862.36 and 880.42 eV are due to the shake-ups of Ni 2p_3/2_ and Ni 2p_1/2_. The Ag 3d peaks showed two separate peaks at 368.15 and 373.44 eV, attributed to Ag 3d_5/2_ and Ag 3d_3/2_. The 6 eV difference between peak’s binding energies is also representative of the metallic silver in the composite [27].

### 3.4. Electrochemical Studies

#### 3.4.1. Electrochemical Studies of PPy, PPy–Ni(OH)_2_, GN@PPy–Ni(OH)_2_, Ag/GN@PPy–Ni(OH)_2_

**Figure 5:**
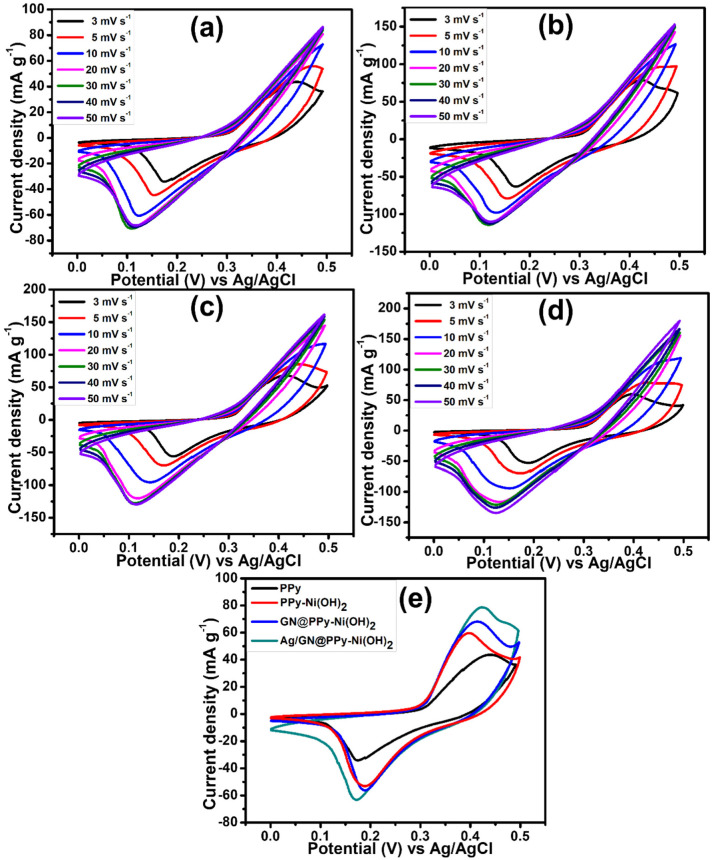
CV curves of (**a**) PPy, (**b**) PPy–Ni(OH)_2_, (**c**) GN@PPy–Ni(OH)_2_, (**d**) Ag/GN@PPy–Ni(OH)_2_, quaternary nanocomposites performed at different scan rates and (**e**) comparative CVs for PPy, PPy–Ni(OH)_2_, GN@PPy–Ni(OH)_2_, and Ag/GN@PPy–Ni(OH)_2_, at 3 mV s^−1^.

The electrochemical characterization of pure PPy and its variants was performed in three electrodes cell using Ag/AgCl as the reference and the platinum wire as a counter, along with a working electrode fabricated by the mechanical coating of active material on nickel foam. Potassium hydroxide was used as an electrolyte (1 M) in all electrochemical measurements. Figure 5a shows the cyclic voltammogram (CV) of PPy at different scan rates from 3 to 50 mV s^−1^ in a potential range of 0–5.0 V. It is evident from the CV that the current density gradually increased as the scan rate increased, showing good electrochemical performance. However, there is a slight shift in redox peak potential at higher scan rates which is associated with the sluggish movement of $OH^{-}$ [28]. Figure 5b shows the CVs obtained for PPy–Ni(OH)_2_ at various scan rates. The addition of nickel hydroxide particles to PPy improved the electrochemical behavior of conducting polymer [29]. The electrochemical studies of GN-wrapped PPy–Ni(OH)_2_ have been presented in Figure 5c. GN is highly conductive: therefore, wrapping of PPy–Ni(OH)_2_ in GN provides the conducting plate form to PPy–Ni(OH)_2_, which promotes the electrochemical performance of ternary composite [30]. The aggregation of electrode material decreases the active sites of electrode material [31]. The introduction of GN into the polymer matrix containing Ni(OH)_2_ particles helps to increase the active sites of GN@PPy–Ni(OH)_2_ nanocomposite. It is well known that a decoration of highly conductive noble metals such as Ag nanoparticles to a nanocomposite material boosts the electrochemical conductivity by providing the pathways to the shuttling of electrons [32]. Figure 5d shows the cyclic voltammograms of the quaternary nanocomposite, Ag/GN@PPy–Ni(OH)_2_. It is clear from the cyclic voltammetry studies that the addition of suitable constituents to the polypyrrole helps to increase the electrochemical performance due to the synergistic effect of all contributing materials in a nanocomposite. Figure 5e provides the comparison studies of pure PPy and its variants at a scan rate of 3 mV s^−1^. A noticeable increase in the current density could be observed, thereby providing evidence of the usefulness of the chosen synthesis strategy for the prepared nanocomposite materials. The specific capacity $\left(Q_{s}\right)$ of the four prepared samples (PPy, PPy–Ni(OH)_2_, GN@PPy–Ni(OH)_2_, Ag/GN@PPy–Ni(OH)_2_) was calculated using the following relation (1) [33];

(1) $$Q_{S}=\frac{1}{\nu m}\int_{Vi}^{Vf}I\times VdV$$

where, ν (V s^−1^) represents the scan rate of cyclic voltammogram, *m* (g) represents the active mass of the material, and the integral part of the relation is the area under the redox peak of CV. The specific capacity values computed at a scan rate of 3 mV s^−1^ for PPy, PPy–Ni(OH)_2_, GN@PPy–Ni(OH)_2_, and Ag/GN@PPy–Ni(OH)_2_ are 134.01, 155.65, 175.20, and 237.25 C g^−1^, respectively.

#### 3.4.2. Galvanostatic Charge–Discharge Studies

The PPy material is considered highly useful for supercapattery because it has superior redox chemistry, large electrical conductivity, and a highly accessible surface area with a specific capacity of 125.85 C g^−1^ in pure form. The PPy was merged with nickel hydroxide, GN, and silver nanoparticles to form binary, ternary, and quaternary nanocomposites for enhanced electrochemical performance for energy storage. The FESEM micrographs, along with the GCD results, provide insight as to how PPy and GN enhance the EC performance and contribute to the phenomena of de−aggregation which in turn, exposed the active sites of the nanocomposite materials.

**Figure 6:**
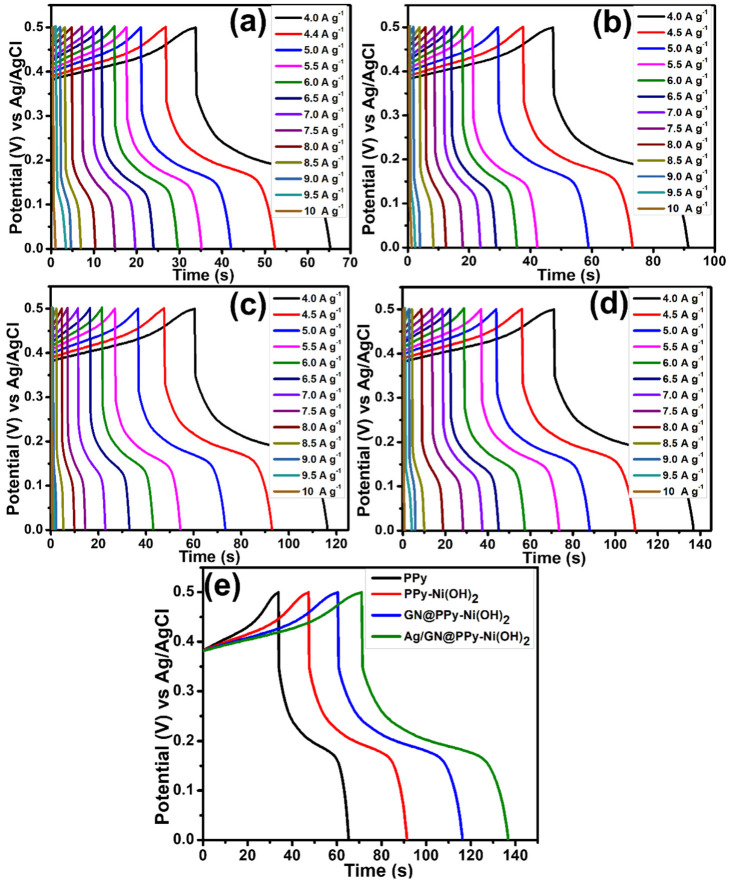
Discharge curves at different current densities: (**a**) PPy, (**b**) PPy–Ni(OH)_2_, (**c**) GN@PPy–Ni(OH)_2_ and (**d**) Ag/GN@PPy–Ni(OH)_2_; and (**e**) comparison of discharge curves of PPy, PPy–Ni(OH)_2_, GN@PPy–Ni(OH)_2_ and (**d**) Ag/GN@PPy–Ni(OH)_2_ at a current density of 4.0 A g^−1^.

The discharge curves of PPy, PPy–Ni(OH)_2_, GN@PPy–Ni(OH)_2_, and Ag/GN@PPy–Ni(OH)_2_ taken in 1 M KOH and galvanostatic current densities between 4.0–10.0 A g^−1^ in Figure 6 indicate that the charging and discharging of the pristine PPy, and its nanocomposites, are completely reversible, which also agrees with the trend of CV. The EC performance of the quaternary nanocomposites (after comparing the discharge curves in Figure 6) is the best out of the four samples owing to the decoration of the Ag nanoparticles and the synergetic impact of Ni(OH)_2_, as well as GN.

The Qs (specific capacity) value of the PPy and its nanocomposites was assessed with the help of the discharge curve through the following expression (2) [34];

(2) $$Qs=\frac{I\times \Delta t}{m}$$

where, *I* (current) is measured in A, Δ*t* (the discharge time) in s and *m* (mass of the electrode material) in *g*. The Qs values found at the current density of 4.0 A g^−1^ were 125.85, 180.48, 228.80, and 268.00 C g^−1^ for PPy, PPy–Ni(OH)_2_, GN@PPy–Ni(OH)_2_, and Ag/GN@PPy–Ni(OH)_2_, respectively.

## 4. Fabrication and Electrochemical Studies of Assembled Energy Storage Device (Supercapattery)

**Figure 7:**
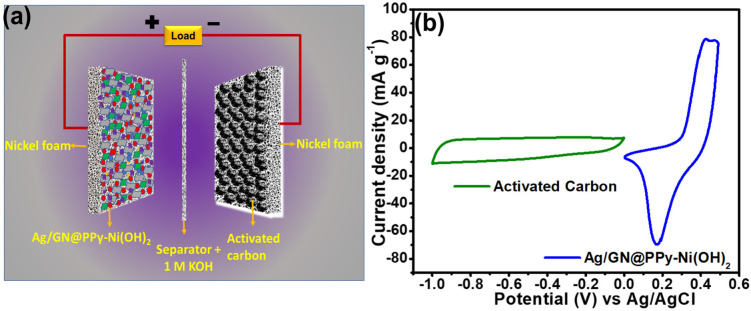
(**a**) Schematic of the constructed supercapattery, (**b**) CVs of AC and Ag/GN@PPy–Ni(OH)_2_ electrodes using a standard three electrodes cell system in 1 M KOH at a scan rate of 10 mV s^−1^.

To get the combined effect of a battery and a capacitor in one device, termed as supercapattery, the battery and capacitive−grade materials were used to construct positive and negative electrodes, respectively. In the present work, Ag/GN@PPy–Ni(OH)_2_ was used as a positive and AC was sued as a negative electrode to build a supercapattery (Ag/GN@PPy–Ni(OH)_2_//AC). The 1 M KOH was used as an electrolyte in ambient conditions for the EC analysis of the constructed device. The schematic of the supercapattery is shown in Figure 7a. The working potential and the electrochemical response of each of the constructed electrode materials (Ag/GN@PPy–Ni(OH)_2_, AC) were tested through separate CVs using a three-electrode cell system before the construction of the device. The potential window for capacitor and battery grade material was −1.0−0 V and 0−0.5 V, respectively (Figure 7b). Thus, if the separate potential of each electrode is combined, the total working potential of the supercapattery was expected to be 1.50 V, which was again reconfirmed through scanning at diverse rates ranging between 0–1.50 V.

**Figure 8:**
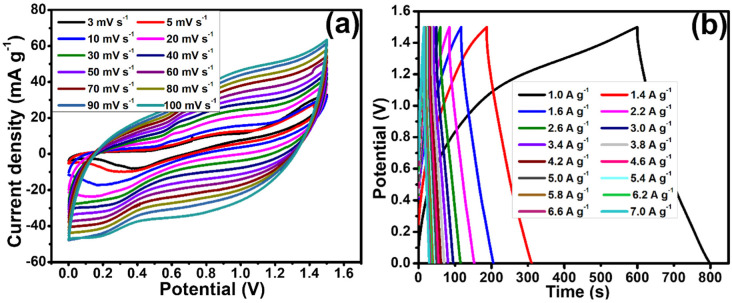
(**a**) CV curves of assembled Ag/GN@PPy–Ni(OH)_2_//AC device assessed in 1 M KOH electrolyte at different scan rates (3−100 mV s^−1^), (**b**) GCD curves at various current densities ranging from 1.0 to 7.0 A g^−1^.

The display of these scan rates ranging from 3–100 mV s^−1^ is revealed in Figure 8a. The pair of distinct redox peaks were seen in CV curves and an increasing current density was also evident with an increase in scan rate. Moreover, the CV shape remained constant even at an elevated scan rate, proving that the constructed device has a good rate capability [35].

Figure 8b displays the GCD profiles of the constructed device at different current densities, which vary from 1.0 to 7.00 A g^−1^ at 1.5 V, a fixed potential. The faradaic reactions during the charging and discharging of the device were evident from the nonlinear GCD curves. An extremely reversible EC trend was seen in the constructed device.

The estimated specific capacity of the device was 211.00 C g^−1^ at 1.0 A g^−1^ by using Equation (1) ($Q_{s}$ = (*I* × ∆*t*)/m). Nevertheless, the $Q_{s}$ was seen to reduce at an increased current density of 7.0 A g^−1^.

**Figure 9:**
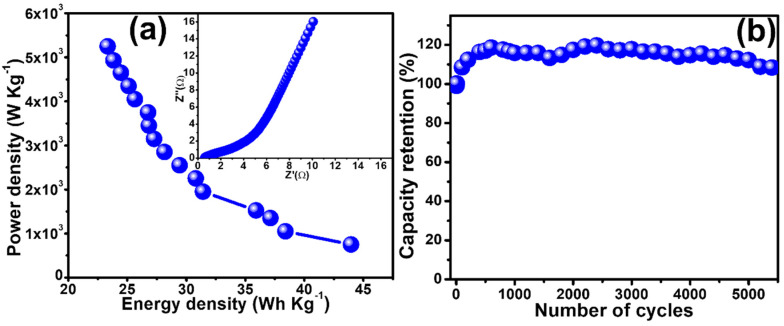
(**a**) Energy density vs. power density graph and inset Figure 9a show the Nyquist plot of EIS studies of assembled device, (**b**) stability studies of the fabricated device, Ag/GN@PPy–Ni(OH)_2_//AC.

The Ragone plot shown in the inset of Figure 9a demonstrates the relation of the energy density, *E*, and power density, *P*, which were calculated by using Equations (3) and (4) [36];

(3) $$E(\mathrm{Wh}/\mathrm{kg})=\frac{\Delta V\times Qs}{2\times 3.6}$$

(4) $$P(W/\mathrm{kg})=\frac{E\times 3600}{\Delta t}$$

where ∆V, the applied potential is taken in *V*, Qs, in C g^−1^ and ∆t, the time required to discharge fully, in seconds (s). The total energy density and the power density acquired by the supercapattery were 20.53 Wh kg^−1^ and 5250.00 W kg^−1^, respectively, at a fixed current density of 7.0 A g^−1^. A decrease from 5250.00 to 750.00 W kg^−1^ in power density was detected when the energy density was changed from 43.96 to 20.53 Wh kg^−1^ with a corresponding current density 7.0 to 1.0 A g^−1^, respectively. The obtained values were higher than those already reported for nanocomposites electrode materials as shown in Table 1. The electrochemical impedance spectroscopy was conducted for the fabricated two electrodes assembly in 1 M KOH. The obtained results are shown in the form of a Nyquist plot in inset Figure 9a. Its high-frequency region shows a semi-circle and a straight line parallel to the y-axis in the low-frequency region, which is an expected behavior for supercapattery. The stability of EC device is an important property which plays a pivotal role in its viability. The retention capacity studies conducted in 1 M KOH electrolyte for 5500 cycles are shown in Figure 9b. During the first 1800 charge–discharge cycles, the capacity behavior of the constructed device grows up, which might be credited to the continuing activation of the electrode material following the enrichment in the perforation of $OH^{-}$ inside the microporous morphology [37]. After that, almost steady behavior was observed that could result from the robust interaction of the constituents over and inside the polymer chain, which might be helpful in capacity retention. That is why an obvious decay in capacity retention was not seen when a study was conducted over a full scale of 5500 cycles; after that a capacity retention of 108.38% was realized [38]. The obtained data support the high stability of quaternary nanocomposites comprising PPy and other contributing constituents, hence making them the right candidates for the fabrication of energy storage devices such as supercapattery.

## 5. Conclusions

In summary, Ag/GN@PPy–Ni(OH)_2_ nanocomposite was prepared by mixing oxidatively polymerized PPy and hydrothermally synthesized Ni(OH)_2_ with GO and Ag precursor and subsequently subjecting it to hydrothermal conditions to get silver decorated and GN wrapped PPy–Ni(OH)_2_ nanocomposite. The morphological analysis showed PPy globules or clusters of Ni(OH)_2_ laked/particles protruding out of PPy globules, along with the GN plus Ag spherical particles. The observance of C, N, O, Ni, and Ag in Ag/GN@PPy–Ni(OH)_2_ from EDAX analysis and their uniform distribution from elemental analysis suggests the efficiency of the synthesis procedure. The structural analysis by XRD and XPS suggests a few layered GN structures and the interaction between the constituents. The electrochemical analysis was done in 1 M KOH using a standard three electrodes cell system, which showed that the quaternary Ag/GN@PPy–Ni(OH)_2_ possessed the highest specific capacity of 237.25 C g^−1^ at 3 mV s^−1^. The synergistic/additional effect of PPy, Ni(OH)_2_, GN, and Ag accounts for the highest electrochemical characteristics of the quaternary composite. The supercapattery assembly of Ag/GN@PPy–Ni(OH)_2_ as a positive and AC negative electrode showed high energy density E = 43.26 Wh kg^−1^ with the associated power density P = 750.00 W kg^−1^ at a current density of 1.0 A g^−1^. The remarkably high energy density of the assembled (Ag/GN@PPy–Ni(OH)_2_//AC) supercapattery showed high cyclic stability after 5500 cycles with a final retention capacity of 108.38% compared to its initial value. The high value of specific capacitance, energy density, and power density, as well as excellent cyclic stability, paves the way for the application of Ag/GN@PPy–Ni(OH)_2_ for high-performance supercapattery.

## Figures and Tables

**Table 1:** Nanocomposite-based electrode materials for energy storage devices.

Electrode Material	Electrolyte	Specific Capacity	Energy Density	Power Density	Ref
Ni_3_(PO4)_2_-Ag_3_PO_4_	1 M KOH	478 C/g	32.4 Wh/kg	399.5 W kg^−1^	[39]
rGO-Co_3_O_4_-Ag	1 M KOH	94.20 C g^−1^	23.63 Wh kg^−1^	440 W kg^−1^	[40]
PANI-SrTiO_3_-250	1 M KOH	63.33 C g^−1^	13.2 Wh kg^−1^	299 W kg^−1^	[38]
PANI-ZnCo_2_O_4_	2 M KOH	398 C g^−1^	13.25 Wh kg^−1^	375 W kg^−1^	[41]
PANI-Ni_3_(PO_4_)_2_-Ag_3_PO_4_	1 M KOH	677 C g^−1^	38.9 Wh kg^−1^	400 W kg^−1^	[42]
Mn-CoS-3/NF	1 M KOH	49.67 F g^−1^	17.96 Wh kg^−1^	806 W kg^−1^	[43]
Ag/Co_3_O_4_@PPy	1 M KOH	355.64 C g^−1^	24.79 Wh kg^−1^	554.40 W kg^−1^	[14]
MWCNT-Co_3_O_4_-Ag	1 M KOH	83.88 C g^−1^	16.5 Wh k g^−1^	297.5 W g^−1^	[44]
Ag/Co_3_O_4_@PANI	1 M KOH	262.62 C g^−1^	14.01 Wh kg^−1^	165.00 W kg^−1^	[45]
NiCoO_2_@CNTs@NF	2 M KOH	151 F g^−1^	56.0 Wh kg^−1^	500.3 W kg^−1^	[46]
MnO_2_/CNF-CNT	1 M Na_2_SO_4_	94.25 F g^−1^	209.4 Wh kg^−1^	1000 W kg^−1^	[47]
PANI/N-CNT@CNT fiber	PVA/ H_3_PO_4_ gel	323.8 F g^−1^ at 1 A g^−1^	5.9 Wh kg^−1^	200 kW kg^−1^	[48]
rGO/CNTs/MnO_2_	1.0 M Na_2_ SO_4_	54.4 F g^−1^ at 0.5 A g^−1^	41.6 Wh kg^−1^	513.7 W kg^−1^	[49]
MnFe_2_O_4_/CNT/ZIF	3 M KOH	330 C g ^−1^	24.4 Wh kg^−1^	265 W kg^−1^	[50]
Ag/GN@PPy-Ni(OH)_2_	1 M KOH	211.00 C g^−1^	43.26 Wh kg^−1^	750.0 W kg^−1^	This Work

## Data Availability

The data presented in this study are available on request from the corresponding author.

## Supplementary Materials

The following supporting information can be downloaded at: https://www.mdpi.com/article/10.3390/polym15051267/s1, Figure S1: TEM images of Ag/GN@PPy–Ni(OH)_2_ nanocomposite at different magnifications.
