# Supplementary material for: Silver Decorated and Graphene Wrapped Polypyrrole@Ni(OH)2 Quaternary Nanocomposite for High Performance Energy Storage Devices
Source: Polymers (Basel). 2023 Mar 2;15(5):1267. doi: 10.3390/polym15051267 (PMC10007114; doi:10.3390/polym15051267)
Supplement: Supplementary file 1 [file polymers-15-01267-s001.zip › polymers-2186563-supplementary.pdf]

polymers

Supplementary Materials

Silver Decorated Graphene Wrapped Polypyrrole@Ni(OH)$_2$ Quaternary Nanocomposite for High Performance Energy Storage Devices

Rashida Jafer [1,*], Sarah A. Alsufyani[1], Javed Iqbal [2] ,Mohammad Omaish Ansari [2,*], Arshid Numan [3,4], Shahid Bashir [5] P. M. Z. Hasan[2] and S.Wageh[1]

1 Department of Physics, Faculty of Science, King Abdulaziz University, Jeddah 21589, Saudi Arabia
2 Center of Nanotechnology, King Abdulaziz University, Jeddah 21589, Saudi Arabia
3 Graphene and Advanced 2D Materials Research Group, School of Engineering and Technology, Sunway University, No.5, Jalan Universiti, Bandar Sunway, Petaling Jaya 47500, Malaysia
4 Sunway Materials Smart Science & Engineering (SMS2E) Research Cluster, Sunway University, No. 5, Jalan Universiti, Bandar Sunway, Petaling Jaya 47500, Malaysia
5 Higher Institution Centre of Excellence (HICoE), UM Power Energy Dedicated Advanced Centre (UMPEDAC), Level 4, Wisma R&D, Universiti Malaya, Jalan Pantai Baharu, Kuala Lumpur 59990, Malaysia
* Correspondence: rmjafer@kau.edu.sa (R.J.); moansari@kau.edu.sa (M.O.A.)

Citation: Jafer, R.; Alsufyani, S.A.; Iqbal, J.; Ansari, M.O.; Numan, A.; Bashir, S.; Hasan, P.M.Z.; Wageh, S. Silver Decorated and Graphene Wrapped Polypyrrole@Ni(OH)$_2$ Quaternary Nanocomposite for High Performance Energy Storage Devices. *Polymers* **2023**, *15*, x. https://doi.org/10.3390/xxxxx

Academic Editor: Guohua Chen

Received: 11 January 2023
Revised: 19 February 2023
Accepted: 27 February 2023
Published: 2 March 2023

Copyright: © 2023 by the authors. Licensee MDPI, Basel, Switzerland. This article is an open access article distributed under the terms and conditions of the Creative Commons Attribution (CC BY) license (https://creativecommons.org/licenses/by/4.0/).

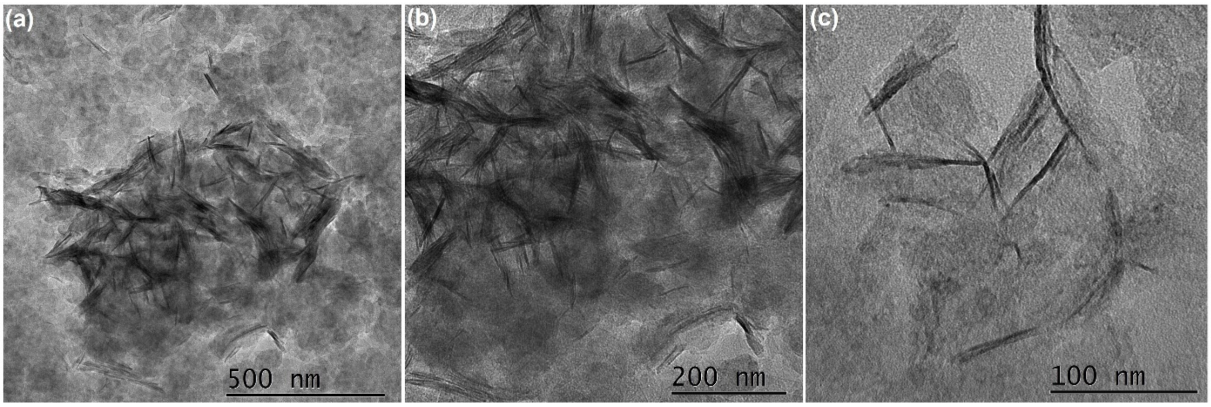

Figure S1. TEM images of Ag/GN@PPy-Ni$(OH)_2$ nanocomposite at different magnifications.
